# Perceived Competence in Nutrition Care Among Hospital Nurses and Its Associated Factors: A Cross-Sectional Study

**DOI:** 10.3390/healthcare14183124

**Published:** 2026-09-21

**Authors:** Sung-Yoon Jo, Kyung-Sook Bang, Eunjung Kim

**Affiliations:** 1 Department of Nursing, Seoul National University Hospital, Seoul 03080, Republic of Korea; josy1997@snu.ac.kr (S.-Y.J.); edema2@snuh.org (E.K.); 2 College of Nursing, Seoul National University, Seoul 03080, Republic of Korea; 3 The Research Institute of Nursing Science, Seoul National University, Seoul 03080, Republic of Korea

**Keywords:** malnutrition, nurses, nutrition, nutrition care competence, nutrition knowledge, nutrition education

## Abstract

**Highlights:**

**What are the main findings?**

- Greater nutrition knowledge, previous nutrition education, longer clinical experience, and working in pediatric units were independently associated with higher perceived nutrition care competency.
- Despite high perceived need for nutrition education and willingness to participate, few nurses had received prior nutrition-related education.

**What are the implications of the main findings?**

- Competency-focused nutrition education may help support nurses in providing nutrition care.
- Clinical settings should foster an environment that promotes the integration of nutrition care into routine nursing practice.

**Abstract:**

**Background/Objectives:** Hospital malnutrition is a prevalent and clinically significant issue associated with prolonged hospital stays, increased complications, and mortality. Although nurses play a key role in monitoring nutritional status and delivering nutrition care, many lack confidence and do not perceive nutrition care as part of their primary responsibilities. This study assessed nurses’ self-perceived nutrition care competence, identified associated factors, and examined their objectively scored nutrition knowledge alongside self-reported nutrition care competence. **Methods:** A cross-sectional study was conducted among unit nurses providing direct patient care at a tertiary hospital in Korea. Data were collected using a structured questionnaire assessing participant characteristics, nutrition education–related characteristics, nutrition knowledge, and self-perceived nutrition care competence. Data were analyzed using descriptive statistics, group comparisons, correlation analyses, and multiple linear regression analyses. **Results:** Among 206 recruited nurses, data from 185 were analyzed. The mean self-perceived nutrition care competence score was 72.24 ± 12.48 (possible range: 24–120). Greater clinical experience, pediatric unit practice, previous nutrition-related education, and higher nutrition knowledge were independently associated with self-perceive nutrition care competence. The mean nutrition knowledge score was 13.81 ± 2.69 (69.1% correct). Notably, although nurses recognized the importance of nutrition care and expressed willingness to receive education, participation in nutrition-related training was limited. **Conclusions:** Self-perceived nutrition care competence was associated with both individual and practice environment factors. Despite the high perceived need for and willingness to participate in nutrition education, previous participation in nutrition-related education was limited. These findings support consideration of educational and organizational strategies to facilitate nutrition care in nursing practice.

## 1. Introduction

Malnutrition is a condition resulting from the insufficient, excessive, or imbalanced intake, absorption, or use of nutrients [1]. However, in hospitalized patients, the term primarily refers to undernutrition [2]. Among them, malnutrition may arise from physiological factors, such as impaired nutrient absorption, and increased losses associated with disease or trauma, and elevated metabolic demands related to illness. Additionally, inadequate nutritional intake attributable to hospitalization-related factors, including mealtime interruptions, meal dissatisfaction, procedure-related fasting, the effects of illness or treatment, and the low prioritization of malnutrition in clinical practice, has been a contributor [3,4].

Concerningly, malnutrition has been reported in more than 40% of hospitalized patients [5]. It is closely associated with higher rates of infection, increased incidence of complications, extended hospitalization, an increased risk of mortality, and increased healthcare costs [5,6,7,8,9]. Accordingly, appropriate nutrition care for hospitalized patients is an integral part of clinical management and essential for improving patient outcomes.

In hospitals, nutrition care encompasses all practices undertaken by healthcare professionals to optimize patients’ nutritional status [10]. Multidisciplinary nutrition care based on collaboration between physicians, nurses, dietitians, pharmacists, and other healthcare professionals is a recommended model for improving health outcomes in hospitalized patients [11]. Specifically, nurses, who maintain the closest and most continuous contact with hospitalized patients, are pivotal throughout the nutrition care process, including monitoring dietary intake, identifying changes in nutritional status, administering enteral and parenteral nutrition, managing nutrition-related complications, and facilitating communication within multidisciplinary teams [12].

However, nurses’ role in nutrition care and the actual implementation of related practices have not been sufficiently established. Nurses have been reported to perceive nutrition care as primarily the responsibility of other healthcare professionals, particularly dietitians [13]. Additionally, nursing students often enter clinical practice without receiving adequate education in nutrition care [14]. This highlights the need for enhanced education and institutional support to strengthen nurses’ nutrition care competencies.

Competency is a multidimensional construct encompassing knowledge, practice, and attitudes. It is a core determinant of healthcare professionals’ abilities to deliver safe and effective interventions [15]. In nursing, nutrition care competency refers to nurses’ ability to effectively deliver nutrition care based on adequate knowledge, nutrition care provision skills, and attitudes supporting such practice [16]. Nurse competency in nutrition care is considered a critical capacity in clinical nursing practice for effectively improving patient nutritional status and health outcomes.

In contemporary healthcare environments, greater emphasis is placed on the assessment and enhancement of practical competencies rather than on knowledge acquisition alone [17]. Accordingly, within the nursing profession, nutrition care competency is increasingly recognized as a key domain of professional competence. However, nutrition care competency has predominantly been assessed using self-report instruments, and previous studies have reported inconsistent findings regarding the usefulness of self-report measures in evaluating nurses’ competencies [18,19]. These conflicting results underscore the need to discuss the applicability and limitations of self-reported competency assessments.

To address these gaps, this study examined clinical nurses’ self-reported nutrition care competency and its associated factors and assessed their nutrition knowledge using an objectively scored measure. The study provides a broader understanding of nurses’ nutrition-related competence along with foundational evidence for developing targeted nutrition education and organizational strategies.

## 2. Materials and Methods

### 2.1. Study Design

This study employed a cross-sectional survey design.

### 2.2. Setting

This study was conducted at a single tertiary-care university hospital that provides inpatient and outpatient services to pediatric and adult populations in Seoul, Republic of Korea. The hospital operates a multidisciplinary Nutrition Support Team (NST) that provides consultation and ongoing monitoring for hospitalized patients who require nutrition support through collaboration among physicians, nurses, dietitians, and pharmacists.

### 2.3. Participants

The participants were unit nurses who provided direct patient care in adult and pediatric inpatient units. Nurses in administrative or managerial positions, who did not engage in direct patient care, were excluded. Additionally, nurses working in specialized clinical settings, including intensive care units, operating rooms, and emergency departments, were excluded owing to differences in clinical roles and care contexts which may limit comparability with general inpatient unit nurses.

The required sample size was calculated a priori using G*Power version 3.1 (Heinrich Heine University Düsseldorf, Düsseldorf, Germany). Following a previous study [20] examining a related component of nutrition care competence, an effect size (f^2^ = 0.119), a significance level of α = 0.05, statistical power of 0.80, and eight independent variables were assumed, yielding a minimum required sample size of 135 participants. To account for an anticipated attrition rate of 20%, the target sample size was set at 165.

### 2.4. Materials

Data were collected using self-administered questionnaires assessing participant characteristics, nutrition education-related characteristics, nutrition knowledge, and self-perceived nutrition care competency. Participant and nutrition education-related characteristics were selected based on factors previously reported to be associated with nutrition care competence among nurses and their clinical relevance to nursing practice [20,21,22,23,24]. Nutrition knowledge and nutrition care competency were measured using previously developed and validated instruments, as described below [16,25].

#### 2.4.1. Participant Characteristics

General and work-related characteristics included sex, age, educational level, total clinical experience, and duration of employment in the current unit and department.

#### 2.4.2. Nutrition Education-Related Characteristics

Nutrition education-related variables included prior participation in continuing or specialized nutrition education programs, perceived need for nutrition education, willingness to participate in future nutrition education, preferred topics for nutrition education, and sources of nutrition-related information.

#### 2.4.3. Nutrition Knowledge

Nutrition knowledge was assessed using a questionnaire developed by Yoon et al., with permission from the authors [25]. The instrument comprises 20 items, including 10 items each assessing general nutrition knowledge and the dietary management of disease conditions. Each item is rated as “true,” “false,” or “don’t know.” Correct responses are scored as 1, whereas incorrect or “don’t know” responses are scored as 0. Total scores range from 0 to 20, with higher scores indicating greater nutrition knowledge. The internal consistency of the instrument was previously reported with a Cronbach’s α of 0.77 [26]. In the present study, the Cronbach’s α was 0.524.

#### 2.4.4. Self-Perceived Nutrition Care Competency

Self-perceived nutrition care competency was assessed using the Nutrition Competence (NUTCOMP) questionnaire, a structured instrument developed to evaluate nutrition-related competencies among primary healthcare professionals [16]. Permission for use was obtained from both the original developer and the Korean version’s developer.

The NUTCOMP questionnaire comprises four primary domains: confidence in nutrition and chronic disease knowledge, confidence in nutrition skills, confidence in nutrition-related communication and counseling, and attitudes toward nutrition care [16]. In this study, the nutrition skills domain was excluded because the specialized nutrition-related tasks it covers are assigned to specially trained clinical dietitians in the study setting and are not typically within the expected scope of practice of unit nurses. Therefore, assessing unit nurses’ confidence in performing these tasks was considered unlikely to adequately reflect their actual clinical roles and responsibilities.

Consequently, the modified NUTCOMP instrument consisted of 24 items rated on a five-point Likert scale. Total scores range from 24 to 120, with higher scores indicating greater self-perceived nutrition care competency. The original instrument demonstrated excellent internal consistency (Cronbach’s α = 0.98) based on all four original domains [16]. Notably, despite excluding the skills domain, the retained domains also demonstrated high internal consistency, with Cronbach’s α values of 0.95 for knowledge, 0.94 for communication and counseling, and 0.88 for attitude [16]. In the present study, Cronbach’s α values were 0.894, 0.947, and 0.836 for the three retained domains, respectively, and 0.928 for the modified total scale.

### 2.5. Data Collection

Data were collected using a self-administered online survey through Google Forms (Google LLC, Mountain View, CA, USA). To minimize the potential for coercion associated with the researcher’s employment at the same institution, recruitment was conducted through the hospital intranet portal without face-to-face contact.

The study information was posted on the hospital’s internal web portal, which was accessible through the institutional intranet. Potential participants reviewed the study purpose, procedures, and participation details via the portal and voluntarily accessed the survey link. The Institutional Review Board approved a waiver of written informed consent. Before beginning the survey, participants reviewed the study information and provided electronic informed consent through Google Forms. Only those who provided consent proceeded to the survey.

Because recruitment was conducted through an intranet announcement without individual contact with potential participants, the exact number of nurses who met the eligibility criteria could not be identified; therefore, the participation and refusal rates could not be calculated. Recruitment and data collection were conducted from 31 October 2025 to 8 January 2026, and recruitment was closed after the prespecified target sample size had been achieved.

### 2.6. Statistical Analysis

Descriptive statistics were used to summarize all variables. Group differences in nutrition care competence were analyzed using independent *t*-tests and one-way analysis of variance. Further, correlations among continuous variables were examined using Pearson’s correlation analysis. Multiple linear regression analysis was performed to identify factors associated with nutrition care competency. Candidate variables were selected based on previous studies, clinical relevance, and univariate associations. Multicollinearity among independent variables was assessed using tolerance and variance inflation factor (VIF) values. Additionally, assumptions of linear regression, including normality and homoscedasticity of residuals, were examined, and the independence of residuals was assessed using the Durbin–Watson statistic. Unstandardized regression coefficients with 95% confidence intervals (CIs) were reported. Statistical analyses were conducted using IBM SPSS Statistics for Mac, version 31.0.1.0 (IBM Corp., Armonk, NY, USA), with statistical significance set at *p* < 0.05.

## 3. Results

### 3.1. Participant Characteristics

A total of 206 nurses participated in the survey. Of these, 21 were excluded because they did not meet the eligibility criteria, including nurses in administrative or educational roles without direct patient care responsibilities and those working outside the target units, such as the emergency department and intensive care unit. Consequently, 185 participants were included in the final analysis (Table 1).

Among the included, the majority were female (93.0%, *n* = 172), with a mean age of 32.05 ± 6.52 years (range, 23–55 years). Notably, 71.9% (*n* = 133) held a bachelor’s degree, 22.7% (*n* = 42) held a master’s degree, and 5.4% (*n* = 10) held a doctoral degree.

Participants reported a mean total clinical experience of 99.63 ± 77.66 months (range, 4–386 months) and mean tenure in their current unit of 45.69 ± 31.87 months (range, 1–240 months). Moreover, 31.9% (*n* = 59) and 68.1% (*n* = 126) worked in pediatric and adult units, respectively.

Notably, only 10.8% (*n* = 20) reported having previously received formal nutrition education, while the remaining had not. Still, the majority perceived a need for nutrition care education in clinical practice (90.8%, *n* = 168) and expressed willingness to participate in such education programs (88.6%, *n* = 164).

The preferred topics for nutrition care education included parenteral nutrition (60.5%, *n* = 112), enteral nutrition (60.0%, *n* = 111), nutrition therapy strategies for specific patient populations (59.5%, *n* = 110), and nutritional assessments (49.2%, *n* = 91). The most frequently reported sources of nutrition knowledge were nurses and other healthcare professionals (49.2%, *n* = 91), followed by in-hospital educational programs (33.5%, *n* = 62), undergraduate education (30.8%, *n* = 57), the Internet or social media (28.6%, *n* = 53), and continuing education (18.4%, *n* = 34). Less commonly reported sources included academic journals or research articles (6.5%, *n* = 12) and conferences (4.3%, *n* = 8).

### 3.2. Nutrition Knowledge Scores and Item Analysis

Table 2 presents the nutrition knowledge scores. The mean score for the basic nutrition knowledge domain (10 items) was 6.45 ± 1.72 (range, 2–10). The mean score for the disease-related dietary knowledge domain (10 items) was 7.36 ± 1.56 (range, 3–10). The overall nutrition knowledge score across all 20 items averaged 13.81 ± 2.69, with observed values ranging from 6 to 19.

An item-level analysis indicated that the highest correct response rates (≥ 90%) were observed for the statements that caffeinated beverages are harmful to ulcer (95.1%); whole grains, fresh vegetables, and seaweed are rich in dietary fiber and help prevent diabetes, hyperlipidemia, and constipation (94.6%); protein is a component of body tissues and blood (94.1%); and a high-protein diet places a burden on the kidneys (91.9%).

Meanwhile, items with correct response rates below 50% included statements that protein and carbohydrates provide the same amount of energy (35.1%); green tea helps reduce halitosis (44.3%); animal protein sources such as cheese, meat, and fish should be restricted to patients with hypertension (44.9%); and fresh fruits and vegetables increase calorie expenditure by helping the body burn other foods (46.5%).

### 3.3. Differences in Self-Perceived Nutrition Care Competency According to Participant and Nutrition Education-Related Characteristics

Table 3 reports the differences in self-perceived nutrition care competency across participants’ characteristics and education-related variables. The mean self-perceived nutrition care competency score for the total sample was 72.24 ± 12.48 out of a possible 120 points.

No statistically significant difference was observed between male (69.85 ± 17.05) and female (72.42 ± 12.11) participants (*t* = −0.718, *p* = 0.474). However, self-perceived nutrition care competency significantly differed by age group (*F* = 3.232, *p* = 0.042), with the highest scores observed among nurses aged 40 years or older (77.15 ± 14.35).

Although nurses with a master’s degree or higher had higher self-perceived competency scores than those with a bachelor’s degree (74.75 ± 11.24 versus 71.26 ± 12.84), this difference did not reach statistical significance (*t* = −1.719, *p* = 0.088).

Significant differences were observed according to the current unit, with nurses working in pediatric units reporting higher self-perceived competency scores than those in adult units (76.36 ± 13.02 versus 70.32 ± 11.78; *t* = 3.141, *p* = 0.002). Additionally, nurses who had received prior nutrition education demonstrated significantly higher self-perceived competency scores than those who had not (80.90 ± 17.39 versus 71.19 ± 11.37; *t* = 2.434, *p* = 0.024).

### 3.4. Correlations Between Self-Perceived Nutrition Care Competency and Related Variables

Table 4 presents the correlations between self-perceived nutrition care competency and related variables. Self-perceived nutrition care competency demonstrated a significant positive correlation with total clinical experience (*r* = 0.161, *p* = 0.029) and nutrition knowledge (*r* = 0.242, *p* < 0.001). Meanwhile, no significant correlation was observed between self-perceived nutrition care competency and the length of experience in the current unit (*r* = −0.015, *p* = 0.836).

### 3.5. Multiple Regression Analysis of Factors Associated with Self-Perceived Nutrition Care Competency 

A multiple linear regression analysis was conducted to identify factors associated with self-perceived nutrition care competency (Table 5). Candidate variables were selected based on previous studies, clinical relevance, and univariate associations. Both age and total clinical experience were significantly associated with self-perceived nutrition care competence in the univariate analyses. As these variables were highly correlated (*r* = 0.974, *p* < 0.001), only total clinical experience was included in the final model because it more directly reflects accumulated professional nursing experience. The Durbin–Watson statistic was 1.951, and tolerance and VIF values ranged from 0.924 to 0.984 and 1.017 to 1.082, respectively, indicating no substantial autocorrelation of residuals or problematic multicollinearity. The regression model was statistically significant (*R*^2^ = 0.179, adjusted *R*^2^ = 0.161, *F* = 9.826, *p* < 0.001).

Higher nutrition knowledge (*β* = 0.201, *p* = 0.004), greater total clinical experience (*β* = 0.167, *p* = 0.019), working in pediatric units (*β* = 0.242, *p* < 0.001), and having received prior nutrition education (*β* = 0.203, *p* = 0.003) were significant associated factors of self-perceived nutrition care competency.

## 4. Discussion

### 4.1. Self-Perceived Nutrition Care Competency Among Clinical Nurses

Nurses’ self-perceived nutrition care competency averaged 72.24 ± 12.48 out of a possible 120 points. This is consistent with studies reporting insufficient or moderate nutrition care competency among nurses [21,27]. For comparisons with international studies using the full NUTCOMP instrument, only the three domains retained in the modified NUTCOMP used in the present study were considered, excluding the nutrition skills domain. Based on these three retained domains, pharmacists and physicians demonstrated higher competency scores than nurses in the present study [28,29]. However, these comparisons should be interpreted with caution because the modified instrument used in this study is not directly comparable with the complete NUTCOMP.

Despite this limitation in direct comparability, the relatively lower self-perceived competency scores observed among nurses are noteworthy. Nurses spend more time with patients than any other healthcare professional. They play a central role in nutrition screening, nutritional status monitoring, patient education, and the implementation of nutrition interventions. Given their central role in nutrition care, the present findings suggest substantial room for improvement in nurses’ self-perceived nutrition care competency.

### 4.2. Factors Associated with Self-Perceived Nutrition Care Competency

Greater clinical experience, working in a pediatric unit setting, nutrition knowledge, and prior nutrition education were identified as significant factors associated with self-perceived nutrition care competency.

Greater clinical experience was associated with higher self-perceived nutrition care competency, possibly reflecting accumulated exposure to nutrition care throughout clinical practice. As nurses gain experience, they encounter a wider range of patients with nutrition-related problems. They also have more opportunities for continued engagement in nutrition care activities, such as nutritional assessment, nutrition-related interventions, and collaboration with multidisciplinary teams. This accumulated clinical exposure may be associated with greater self-perceived nutrition care competency. However, two studies found no significant association among nurses [21,27], while another study reported that greater clinical experience was significantly associated with more positive attitudes toward nutrition support and higher nutrition-related skills [22]. These discrepancies may reflect differences in clinical settings and role expectations among nursing populations. Nevertheless, the present findings suggest that clinical experience may be an important factor associated with nurses’ self-perceived nutrition care competency.

Besides clinical experience, the current practice setting was also identified as a significant factor associated with self-perceived nutrition care competency. Nurses working in pediatric units demonstrated significantly higher self-perceived nutrition care competency than those working in adult units. This finding may partly reflect the distinct physiological and nutritional needs of pediatric patients. Nutritional requirements vary substantially according to age and developmental stage [30]. Specifically, children have smaller energy reserves and higher metabolic demands than adults, making them more vulnerable to nutritional deprivation [30]. Moreover, children have a higher proportion of body water and a larger surface area relative to body size than adults, making fluid imbalances more common and necessitating careful monitoring of fluid and electrolyte status [31]. Thus, nutritional assessment is an integral component of pediatric care, particularly for children with acute and chronic diseases [32]. Accordingly, appropriate nutritional provision is a key performance indicator in pediatric nursing, with particular attention to meeting age-specific nutritional requirements to support growth and development [33,34]. These characteristics may provide pediatric nurses with greater exposure to nutrition care and may partly explain their higher perceived nutrition care competency in this study.

This finding is consistent with studies reporting nutrition care competency differences by clinical practice setting. Higher nutrition care competency has been observed among nurses working in intensive care, nephrology, and hematology-oncology units compared with those working in other clinical areas [21,27]. This may be because nurses in these settings may more frequently encounter nutrition-related issues. Thus, they may have greater opportunities to engage in nutritional assessment, monitoring, and intervention as part of routine patient care. The present findings suggest that continued exposure to clinical environments that routinely prioritize nutrition management may be associated with higher self-perceived nutrition care competency.

However, the association between pediatric unit practice and self-perceived nutrition care competency should be interpreted with caution. Working in a pediatric unit may serve as a proxy for unmeasured institutional and clinical factors rather than reflecting an independent effect of the practice setting itself. For example, pediatric and adult units may differ in the frequency of interactions with the NST, multidisciplinary collaboration in nutrition care, nursing workload, and patient care complexity. As these factors were not measured in the present study, their potential contribution to the observed association could not be determined.

Next, nutrition knowledge emerged as a significant factor associated with self-perceived nutrition care competency. This aligns with the conceptualization of competency as an integration of knowledge, skills, and attitudes. Essentially, adequate nutrition knowledge may provide an important foundation for the development and application of nutrition care competency in clinical practice [15].

The mean nutrition knowledge score in this study corresponded to 69.1% correct responses, higher than the 58.4–61.6% reported in previous studies [22,35,36]. Despite this relatively higher overall score, performance varied considerably across individual items, with correct response rates ranging from 35.1% to 95.1%. Interestingly, the item-level analysis revealed higher accuracy on questions related to dietary management of disease conditions than on those assessing general nutrition knowledge. Similar findings have been reported, with nurses demonstrating better performance on clinically relevant nutrition questions than on general nutrition knowledge items [23]. This pattern may reflect repeated exposure to disease-specific nutrition issues in clinical practice, through which nutrition-related knowledge is reinforced over time. Meanwhile, foundational nutrition knowledge may receive less emphasis after undergraduate education and may not be consistently reinforced in routine clinical settings.

Further, nutrition education was significantly associated with self-perceived nutrition care competency, whereas no significant differences were observed according to formal education level. This is consistent with studies reporting that nurses who had received nutrition-related education demonstrated more positive attitudes toward nutrition care [20,22] and higher nutrition care competencies [21,24,27]. Evidently, nutrition-specific educational experiences may be more strongly associated with self-perceived nutrition care competency than formal academic degrees alone.

Notably, despite the limited proportion of nurses who had received nutrition-related education, the perceived need for nutrition education and willingness to participate in future educational programs were high. This discrepancy highlights a gap between nurses’ educational needs and available learning opportunities. As such, current educational provision may be insufficient to meet clinical nurses’ growing demand for nutrition-related learning. The high preference for enteral and parenteral nutrition as educational topics further suggests that nurses are seeking practical knowledge, which can be readily applied in everyday clinical practice.

Moreover, nurses and other healthcare professionals were identified as the most frequently reported sources of nutrition knowledge, whereas formal educational opportunities and evidence-based resources were reported less frequently. This indicates that, in the absence of sufficient formal educational opportunities, nurses may primarily rely on workplace-based experiential learning to develop nutrition knowledge rather than through structured educational programs or evidence-based learning resources.

Overall, these findings are consistent with research suggesting that, although the importance of nutrition in the management of chronic diseases has increasingly been emphasized, nutrition education for healthcare professionals remains insufficient [37].

### 4.3. Nursing Education and Practice Implications

Supporting nutrition care among clinical nurses may require both educational and organizational strategies. Although undergraduate nursing education provides an essential foundation, it cannot fully address the continuously evolving body of nutrition knowledge required in contemporary healthcare [37]. Therefore, nutrition education should not be limited to undergraduate programs but should be provided throughout nurses’ professional careers, including graduate education, hospital-based education, and continuing professional development, to provide up-to-date, evidence-based knowledge and support its application in clinical decision-making and practice. In this context, and consistent with the American Association of Colleges of Nursing Essentials, competency-based educational approaches that emphasize active, experiential, and collaborative learning, including case-based discussions, simulation-based learning, and team-based learning, may be particularly effective in strengthening nutrition care competency [17,38].

However, educational strategies alone may not fully address the contextual factors relevant to nutrition care competency. Nutrition care is increasingly considered important, with growing evidence demonstrating its benefits for patient outcomes [39]. However, nutrition-related activities continue to be perceived as secondary responsibilities in many clinical settings [4,10,22]. Recent qualitative evidence has further identified competing workloads, limited organizational emphasis, lack of standardized protocols, and insufficient interprofessional collaboration as barriers to integrating nutrition care into routine clinical practice [4]. The findings also suggest that practice environment may be relevant to self-perceived nutrition care competency, as nurses working in pediatric units demonstrated significantly higher self-perceived nutrition care competency than those working in adult units. This association may partly reflect differences in exposure to nutrition-focused clinical practice. Therefore, hospitals should foster clinical environments wherein nutrition care is recognized as a core component of nursing practice and is routinely supported in patient care.

At the organizational level, current clinical guidelines recommend that all hospitalized patients undergo nutrition screening within 24 h of admission and emphasize the important role of multidisciplinary collaboration in delivering high-quality nutrition care [40,41]. In this context, NSTs may serve not only to coordinate nutrition care but also provide valuable opportunities for workplace-based learning, interprofessional collaboration, and the dissemination of evidence-based nutrition practices.

In addition, hospitals may consider incorporating nutrition care competency into orientation programs for newly employed nurses, career ladder programs for experienced nurses, and ongoing institutional competency development initiatives. Overall, integrating educational and organizational strategies may provide a supportive foundation for incorporating nutrition care into routine nursing practice and promoting the provision of high-quality nutrition care.

### 4.4. Strengths and Limitations

This study has several strengths. To our knowledge, this is one of the few studies to examine self-perceived nutrition care competency along with objectively scored nutrition knowledge among hospital nurses. By examining these measures together, this study provides complementary information on nurses’ nutrition knowledge and self-perceived nutrition care competency. Furthermore, this study identified factors associated with self-perceived nutrition care competency, including clinical experience, work unit, previous nutrition education, and nutrition knowledge. These findings may inform the development of tailored educational and organizational strategies that support nutrition care in different clinical settings.

Nevertheless, this study has several limitations. First, its cross-sectional design precludes causal inferences regarding the study variables. Second, this study was conducted at a single tertiary hospital in Seoul, Republic of Korea, and included only nurses working in adult and pediatric general inpatient units. Therefore, the findings may have limited generalizability to nurses working in other regions, hospital settings, and clinical specialties. Future multicenter studies involving nurses from diverse regions, hospital types, and clinical settings are needed to assess the generalizability of these findings. Third, the regression model’s explanatory power was relatively modest, suggesting that relevant organizational and contextual factors may not have been fully captured. Future research should examine factors such as the frequency of interactions with the NST, multidisciplinary collaboration in nutrition care, nursing workload, and patient care complexity.

Fourth, the modified NUTCOMP used in this study excluded the nutrition skills domain. The content validity, construct validity, and factor structure of this three-domain, 24-item adaptation were not established in the present study, and its utility across different professional groups or hospital settings remains unknown. Accordingly, the findings should be interpreted with caution, particularly regarding the comprehensive assessment of self-perceived nutrition care competency and direct comparability with studies using the full NUTCOMP.

Fifth, the nutrition knowledge instrument showed relatively low internal consistency in the present sample; therefore, the nutrition knowledge scores should be interpreted with caution. In addition, the instrument primarily assessed general and disease-related nutrition concepts and may not adequately capture knowledge relevant to current hospital nutrition care, including enteral and parenteral nutrition, nutrition screening, and clinical decision-making. The low internal consistency and limited clinical scope of the instrument may have introduced measurement error, potentially affecting the observed association between nutrition knowledge and self-perceived nutrition care competency. Future studies should use nutrition knowledge instruments specifically developed or clinically validated for hospital nurses.

Finally, voluntary intranet-based recruitment may have introduced self-selection bias, as nurses with greater interest in nutrition and nutrition care may be more likely to participate. Because the exact number of eligible nurses was unavailable, the participation and refusal rates could not be determined, limiting the assessment of sample representativeness. Although the final sample exceeded the minimum sample size required by the a priori power analysis, this statistical adequacy does not ensure sample representativeness. Additionally, the data were collected using a self-reported questionnaire, and self-perceived nutrition care competency was not verified through clinical observation, objective performance evaluation, or patient outcomes. Therefore, the findings may be subject to response bias, including social desirability bias, and may not fully reflect participants’ actual clinical performance.

## 5. Conclusions

This study evaluated nurses’ nutrition knowledge and self-perceived nutrition care competency and identified factors associated with self-perceived nutrition care competency in clinical practice. Clinical experience, pediatric unit practice, prior nutrition education, and nutrition knowledge were significantly associated with self-perceived nutrition care competency.

Although nurses recognized the importance of nutrition care and expressed a strong willingness to participate in educational programs, their previous exposure to nutrition-related education was limited. These findings highlight the need for competency-based nutrition education and supportive clinical environments to support nutrition care into routine nursing practice.

## Figures and Tables

**Table 1:** Participant characteristics.

Variable		*n* (%) or M ± SD	Range
Sex	Male	13 (7.0)	
	Female	172 (93.0)	
Age (years)		32.05 ± 6.52	23–55
Education level	Bachelor’s degree	133 (71.9)	
	Master’s degree	42 (22.7)	
	Doctoral degree	10 (5.4)	
Total clinical experience (months)		99.63 ± 77.66	4–386
Experience in current unit (months)		45.69 ± 31.87	1–240
Current unit	Pediatric unit	59 (31.9)	
	Adult unit	126 (68.1)	
Previous nutrition education	Yes	20 (10.8)	
	No	165 (89.2)	
Perceived need for nutrition care education in clinical practice	Yes	168 (90.8)	
	No	17 (9.2)	
Willingness to participate in nutrition care education	Yes	164 (88.6)	
	No	21 (11.4)	
Preferred topics for nutrition care education *	Enteral nutrition	111 (60.0)	
	Parenteral nutrition	112 (60.5)	
	Nutritional assessment	91 (49.2)	
	Nutrition therapy strategies for specific patient populations	110 (59.5)	
Sources of nutrition knowledge *	Undergraduate education	57 (30.8)	
	In-hospital education programs	62 (33.5)	
	Continuing education	34 (18.4)	
	Conferences	8 (4.3)	
	Academic journals or research articles	12 (6.5)	
	Nurses and other healthcare professionals	91 (49.2)	
	Internet or social media	53 (28.6)	

* Multiple responses were allowed.

**Table 2:** Nutrition knowledge scores and item-level correctness.

Domain	Item	Correct *n* (%)
Basic Nutrition Knowledge (10 items)	* Protein and carbohydrates provide the same amount of energy (calories).	65 (35.1)
	To lose weight, fat should be eliminated from the diet.	144 (77.8)
	* Fresh fruits and vegetables increase calorie expenditure by helping the body burn other foods.	86 (46.5)
	* Beans are a good source of dietary fiber.	154 (83.2)
	Vitamins and minerals are nutrients that supply energy.	132 (71.4)
	* Cholesterol is required for the synthesis of steroid hormones and bile acids.	98 (53.0)
	Desirable weight loss is losing 3 kg per week.	157 (84.9)
	* Protein is a component of body tissues and blood.	174 (94.1)
	* Green tea helps reduce halitosis (bad breath).	82 (44.3)
	* Alcohol provides more calories than sugar.	101 (54.6)
	Subtotal score, mean ± SD (range)	6.45 ± 1.72 (2–10)
Disease-related dietary knowledge (10 items)	* Raisins, dried persimmons, egg yolk, oysters, and liver are beneficial for preventing anemia.	129 (69.7)
	* Whole grains, fresh vegetables, and seaweed are rich in dietary fiber and help prevent diabetes, hyperlipidemia, and constipation.	175 (94.6)
	* Caffeinated beverages are harmful for ulcer.	176 (95.1)
	Pork, chicken, and beef are beneficial for preventing atherosclerosis.	145 (78.4)
	Animal protein sources such as cheese, meat, and fish should be restricted in patients with hypertension.	83 (44.9)
	Bread, noodles, and white rice are unnecessary for patients with diabetes, because carbohydrate intake should be restricted.	114 (61.6)
	* A high protein diet places a burden on the kidneys.	170 (91.9)
	* Fresh fruits and vegetables are beneficial for anemia.	128 (69.2)
	* Liver, egg yolk, and dried mushrooms help prevent osteoporosis.	105 (56.8)
	Frequent consumption of smoked and salted foods has no association with cancer risk.	137 (74.1)
	Subtotal score, mean ± SD (range)	7.36 ± 1.56 (3–10)
Total nutrition knowledge (20 items)	Total score, mean ± SD (range)	13.81 ± 2.69 (6–19)

* Items with a correct answer of “Yes”.

**Table 3:** Self-perceived nutrition care competency by participant characteristics.

Variable		*n*	Mean ± SD	*t*/*F* (*p*)
Total		185	72.24 ± 12.48	-
Sex	Male	13	69.85 ± 17.05	−0.718 (0.474)
	Female	172	72.42 ± 12.11	
Age (years)	<30	78	70.21 ± 10.92	3.232 (0.042)
	30≥, <40	80	72.58 ± 12.89	
	40≥	27	77.15 ± 14.35	
Education level	Bachelor’s degree	133	71.26 ± 12.84	−1.719 (0.088)
	Master’s degree or higher	52	74.75 ± 11.24	
Current unit	Pediatric unit	59	76.36 ± 13.02	3.141 (0.002)
	Adult unit	126	70.32 ± 11.78	
Previous nutrition education	Yes	20	80.90 ± 17.39	2.434 (0.024)
	No	165	71.19 ± 11.37	

**Table 4:** Correlations between self-perceived nutrition competence, clinical experience, and knowledge.

		Total Clinical Experience	Experience in Current Unit	Nutrition Knowledge
Self-perceived nutrition care competency	Pearson correlation	0.161	−0.015	0.242
	*p*-value (two-tailed)	0.029	0.836	<0.001
	*N*	185	185	185

**Table 5:** Multiple regression analysis of factors associated with self-perceived nutrition care competency.

Variables	*B*	95% CI for *B*		SE	β	*t*	p	VIF
		Lower	Upper					
Constant	53.741	44.915	62.566	4.473		12.016	<0.001	
Nutrition knowledge	0.934	0.308	1.560	0.317	0.201	2.944	0.004	1.023
Total clinical experience	0.027	0.005	0.049	0.011	0.167	2.374	0.019	1.082
Current unit	6.451	2.788	10.114	1.856	0.242	3.475	<0.001	1.060
Previous nutrition education	8.133	2.749	13.517	2.728	0.203	2.981	0.003	1.017

*B* = unstandardized coefficient; CI = confidence interval; SE = standard error; *β* = standardized coefficient; VIF = variance inflation factor. Current unit (1 = pediatric unit, 0 = adult unit); previous nutrition education (1 = yes, 0 = no).

## Data Availability

The data are available on reasonable request from the corresponding author. The data are not publicly available due to ethical and privacy restrictions related to participant confidentiality.

## Abbreviations

The following abbreviations are used in this manuscript:

NUTCOMP	Nutrition Competence
NST	Nutrition Support Team
